# Linking Human Perceptions and Spotted Hyena Behavior in Urban Areas of Ethiopia

**DOI:** 10.3390/ani10122400

**Published:** 2020-12-15

**Authors:** Julie K. Young, D. Layne Coppock, Jacopo A. Baggio, Kerry A. Rood, Gidey Yirga

**Affiliations:** 1 USDA-National Wildlife Research Center, Predator Research Facility, Millville, UT 84326, USA; 2 Department of Environment and Society, Quinney College of Natural Resources, Utah State University, 5215 Old Main Hill, Logan, UT 84322, USA; layne.coppock@usu.edu; 3 School of Politics, Security, and International Affairs, National Center for Integrated Coastal Research (UCF Coastal), University of Central Florida, 4297 Andromeda Loop N. Howard Phillips Hall, Orlando, FL 32816, USA; jacopo.baggio@ucf.edu; 4 Animal Dairy and Veterinary Sciences Department, Utah State University, 4815 Old Main Hill, Logan, UT 84322, USA; kerry.rood@usu.edu; 5 Department of Biology, Mekelle University, P.O. Box 231, Mekelle, Tigray, Ethiopia; gidey.yirga@yahoo.com

**Keywords:** animal behavior, flight initiation distance test, focus group interview, human perceptions, key informant interview, puzzle box

## Abstract

**Simple Summary:**

Humans and carnivores are co-occurring in many landscapes and especially in urban areas. This can result in increased tolerance or conflict, pending the perceptions by humans and behaviors of both species. Here, we provided a case study of how data can be collected on both humans and carnivores in areas where they co-occur to provide foundational information to understand potential linkages and feedback loops between the two. We did so by obtaining data on spotted hyenas and people living in several urban areas of Ethiopia.

**Abstract:**

Humans have shaped carnivore behavior since at least the Middle Paleolithic period, about 42,000 years ago. In more recent times, spotted hyenas (*Crocuta crocuta*) in Ethiopia have adapted to living in urban areas, while humans have adapted to living with hyenas. Yet, relationships between coexisting humans and carnivores are rarely addressed beyond mitigating conflicts. We provided a case study for how to broadly think about coexistence and how to study it when measuring if humans and carnivores affect one another. We collected data in four Ethiopian cities: Mekelle, Harar, Addis Ababa, and Arba Minch. We held focus groups and key informant interviews that incorporated feedback from 163 people, representing a wide array of religious, economic, and educational backgrounds. We also determined how many hyenas resided in these cities, hyena behavioral responses to humans using a flight initiation test, and problem-solving abilities via puzzle box trials. We found that in three of the cities, hyenas and humans coexist at high densities and frequently encounter each other. While all participants recognized the importance of hyenas as scavengers to maintain a clean environment, there was pronounced variation in cultural perspectives across cities. For example, while the people of Harar revere hyenas in spiritual terms, in Arba Minch hyenas were regarded as nuisance animals. Hyenas were universally respected as a formidable predator across cities but reports of attacks on livestock and humans were few. Flight initiation tests revealed hyenas fled at significantly closer distances in Harar and Addis Ababa than in Mekelle. Hyenas succeeded at solving a puzzle box in Harar but not in Mekelle. These variable behavior in hyenas correlated to different human perceptions. Our case study results suggest that the hyena–human dynamic is highly variable across these locations. We conclude by exploring the implications of these findings for how humans and hyenas can shape one another’s behavior. Developing studies to link human perceptions and animal behavior could advance wildlife conservation, especially in urban areas.

## 1. Introduction

By 2050, 68% of the estimated 8.5 billion people on earth will reside in urban areas [1], while large carnivores will have lost another 10–25% of their native habitat [2]. Urban sprawl will continue to encroach on wildlife habitats and increase the probability of contact among wildlife, humans, and domestic animals [3]. Further, increased urbanization may facilitate the loss of connectivity between people and nature [4], which has clear consequences on potential human–carnivore interactions. While humans have shaped carnivore behavior since the Middle Paleolithic [5], recent global examples abound of a rapidly emerging scenario where large carnivores coexist with humans in densely populated urban settings and humans likely have strong effects on carnivore behavior [6]. This realignment of human–carnivore urban interactions is occurring across six continents, including coyotes (*Canis latrans*) in North America [3], leopards (*Panthera pardus*) in India [7], brown bears (*Ursus arctos*) in Eastern Europe [8], jaguarondi (*Puma yagouaroundi*) in South America [9], and spotted hyenas (*Crocuta crocuta*) in Africa [10].

Humans provide reliable and predictable food subsidies that can improve individual fitness of the wildlife utilizing resources found in urban settings [11,12]. Urban carnivores feed on livestock offal, human waste, and other forms of organic matter in household refuse [13,14]. Urban food resources are often clumped in areas that increase the probability of closer interactions with humans and conspecifics. This proximity could reduce their fear of humans (e.g., [15]) and increase bold and aggressive behavior by carnivores [16]. Urban areas may provide refugia from humans, because there is often a ban on hunting, and from other dominant or competing carnivores that are only found outside of urban zones. 

Carnivores, on the other hand, affect several aspects of human welfare. Livestock deaths represent losses of capital assets, leading to wildlife–human conflict and negative perceptions among livestock owners regarding large carnivores (e.g., [17]). Attacks on humans and their pets may increase in relation to increased use of urban areas by carnivores [18]. However, not all interactions between large carnivores and people are negative [19]. Large carnivores can provide ecosystem services (e.g., [20]), generate revenue for hunting and eco-tourism enterprises [21,22,23], indirectly reduce the scope of disease proliferation [24], or directly mitigate disease-vector risks to human health [25]. Citizens in all societies can also have strong emotional ties with large carnivores. Positive support for large carnivore re-introductions in American or European wildlands, for example, is common among urban populations [26]. Positive connections with animals may also be linked to improved mental health and social resilience, the ability of a human society to recover from a disturbance or challenge among communities [27]. In contrast, however, carnivore attacks on people can have very negative impacts on the mental health of survivors and their family members [28]. Large carnivores play diverse roles in human culture (e.g., [27,29]).

Interactions between urbanites and carnivores are affected by the perceptions and attitudes of humans, as well as potential changes in behavior exhibited by carnivores. Yet, most studies either evaluate human perceptions and behaviors (e.g., [30]) or carnivore population ecology and behavior (e.g., [31]), with limited effort to examine linkages among the two components (but see [32]). Here, we presented a case study illustrating how data can be collected in urban areas of Ethiopia on both the behavior of a large urban carnivore, the spotted hyena, and human perceptions towards hyenas. In Ethiopia, households lose approximately 3% of their herds in any given year to hyena predation [33,34], but hyenas that live in cities survive off of human refuse and offal [14] and attract tourism [35]. Studies from throughout their range show spotted hyenas can shift their diet [14], foraging behavior [36], behavioral patterns [37], and personality [38] in response to human-induced and natural changes to their environment. Using urban-dwelling hyenas in Ethiopian cities as a model system, our objective was to provide a case study for how human and carnivore data can complement one another to assess the relationship between humans and carnivores where they coexist. We suspect that hyenas will show more bold and exploratory behaviors in areas where humans have more positive perceptions and behaviors. We used this case study as an exploratory attempt to determine if complimentary human and hyena data can be obtained from the same locations and whether correlations between the two types of data exist. 

We first obtained information on human beliefs, perceptions, and behaviors relevant to hyenas by conducting focus group discussions [39] and key informant interviews [40]. This provided the foundation of a conceptual model of humans’ perceptions and beliefs in urban dominated settings for hyenas with emphasis on human livelihoods, culture, and health. At the same time, we determined the presence of spotted hyenas in the urban areas using playback surveys (e.g., [41]). If hyenas were found to exist in the urban area, we then obtained information on urban hyena behavior via a puzzle box test (e.g., [42]) and flight initiation distance test [43]. These techniques were used to assess behavioral patterns and cognition of hyenas in response to living in urban systems. Finally, we jointly assessed attributes of hyena behavior and human attitudes, behavior, and perception of hyenas to highlight potential feedback loops between the perception and behavior of humans and hyenas. Our case study of human–carnivore relationships highlights the utility of conducting such research through the complementary lenses of multiple disciplines.

## 2. Materials and Methods

**Figure 1:**
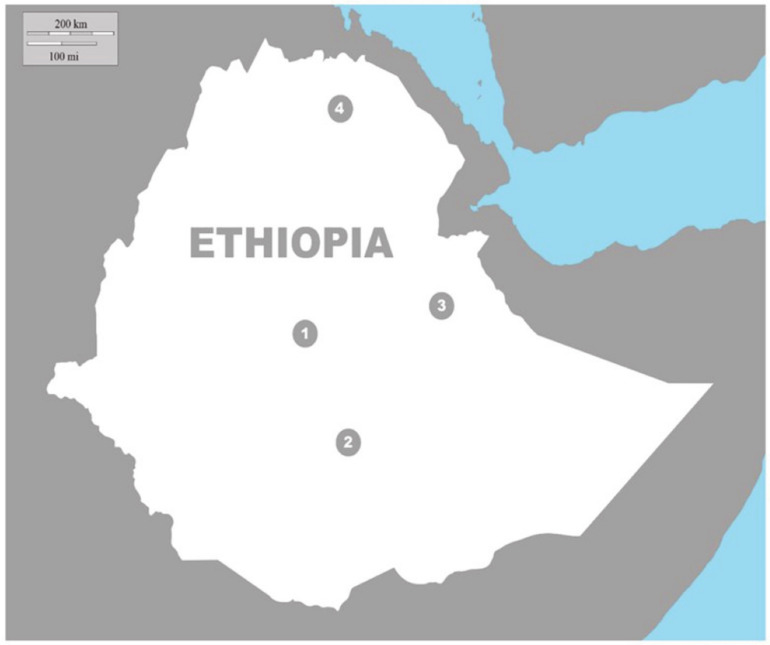
Location of four cities (1: Addis Ababa; 2: Arba Minch; 3: Harar; and 4: Mekelle) in Ethiopia where data were collected on spotted hyenas (1, 3, 4) and human perceptions and beliefs related to humans (2, 3, 4). Figure courtesy of B. Kartchner, Utah State University.

To provide a case study on hyena–human interactions, we collected all field data from humans and hyenas between 2 August and 12 September 2017. We obtained these data from four urban centers in Ethiopia: Harar, Mekelle, Addis Ababa, and Arba Minch (Figure 1). These urban centers are characterized by a gradient of hyena’s cultural significance, land-use change, extent of peri-urban landscapes, and economic development. They were selected based on previous research on urban hyenas (Harar and Mekelle) and conversations with Ethiopian colleagues on which additional cities may have resident hyenas (Addis Ababa and Arba Minch). While other cities in Ethiopia may also have resident hyenas, these cities were expected to have abundant hyena populations and represented a wide range of geographical locations and human densities that were logistically possible for us to visit in the time frame of our case study. 

Harar has been designated as a United Nations Educational, Scientific, and Cultural Organization (UNESCO) World Heritage site and is among the top Ethiopian destinations for international and domestic tourists; formal hyena-feeding exhibitions are a prominent draw for visitors. It is the only city of the four we studied for which information already existed on the relationship between humans and hyenas. Hyenas have long been an important part of the local culture, environment, and economy [21]. There is a widely held view that hyenas in Harar consume evil spirits (“jinn”) at night and are thus spiritual guardians of the city [35]. 

The vicinity of Mekelle supports extensive, cultivated lands for cereal production, livestock grazing areas, hilltop villages, church forests, and large expanses of hillsides under recent protection from livestock grazing to promote environmental rehabilitation. People mainly see hyenas in and around Mekelle in the evenings when hyenas are foraging in the urban city center and at the city landfill. 

The city of Addis Ababa occurs in North-Central Ethiopia and serves as the national federal capital. The landscape around Addis Ababa includes mountains, hills, open plains, and numerous river channels that penetrate the city. People commonly see and hear hyenas in Addis Ababa at night. 

The city of Arba Minch is one of the largest cities in Southwestern Ethiopia. It is in the Rift Valley. The landscape around Arba Minch is generally flat and dominated on three sides by intensive agriculture including cereal grains and fruit groves. On the east side of the city are steep slopes that descend into Nech Sar National Park; the park contains gallery forests and an open savanna and occupies the isthmus between Lakes Chamo and Abaya. Hyenas are occasionally seen on the outskirts of Arba Minch, but they reside in the park.

### 2.1. Human Survey Data Collection

Information was collected from adult Ethiopians who were at least 18 years old using focus group discussions and key informant interviews (hereafter referred to as interviews). Research protocols and permissions were approved by Institutional Review Boards (IRBs) and other authorities at Utah State University and Mekelle University in Ethiopia (USU IRB: 8662). No human information was collected for the Addis Ababa study area because we were unable to get social research pre-approved by local administrators. We collected information over four days in Arba Minch and six days each in Harar and Mekelle. 

Because the time per location was very limited, information was collected opportunistically. We began with the intent to engage a wide array of people at each study site as potentially distinguished by age, gender, religious affiliation, wealth class, formal education, and connectivity to public service. We expected to engage the general public via focus groups and use interviews for public servants and others likely to possess professional or policy-related viewpoints concerning hyena issues. Upon arrival in each study site, we first contacted individuals who were likely to serve as field assistants (roles described below), some of whom had been pre-identified in the design phase based on e-mail advice from academic contacts, while in one case (Arba Minch) we lacked such contacts and went directly to the local university administration to identify suitable candidates. The key attributes of field assistants were (1) diverse language skills, (2) general knowledge of hyena–human interactions in the locality, and (3) ability to scour their social networks to rapidly engage a diverse group of people. It was the field assistants, as directed by the team social scientist, who quickly identified candidates for focus groups and key informant interviews. Some focus group discussions and interviews were formally scheduled with a lead time of one or two days, while others were spontaneous depending on who was encountered in mutually favorable situations.

Letters of information (LOI) that described the research purpose, as well as the risks and benefits to participants, were given to all potential recruits in their local language. If potential recruits were illiterate, LOIs were read aloud face-to-face. Informed consent was procured from all people who participated. Cash incentives were used to encourage participation and averaged at 5 USD per person, a rate advised by local members of our research team. Government officials declined payments. 

Implementation of focus group discussions and interviews followed standard procedures [39,40]. The focus group discussions were intended to capture views from a diverse array of citizens, while interviews were directed at government officials, community leaders, and highly educated professionals. Information capture for both focus group discussions and interviews relied on synchronous, hand-written notetaking on field forms with a different field assistant for each location due to variation in the dominant languages across sites. Each field assistant was trained by the team social scientist to serve multiple roles as a translator, focus group moderator, and key informant interviewer. No audio recordings were employed as Ethiopians in general tend to be averse of this practice (D. L. Coppock, personal observation). The focus group discussion and interview information were first transcribed in local languages and then translated to English for final summary interpretations. Field assistants were largely fluent in English and local languages including Amharic (Arba Minch), Harari and Amharic (Harar), or Tigrinya (Mekelle). Because of variation in languages, translations, and interviewer skills—as well as the lack of audio recordings—data collection did not attempt to capture specific quotations from respondents.

The focus group discussions and interviews relied on the same core list of five, open-ended questions: (1) Have you had direct personal experiences with hyenas; (2) have you indirectly learned about hyenas from other people; (3) what are your personal opinions about hyenas; (4) what are your opinions about hyena management; and (5) is there anything important about hyenas that has not been mentioned. In each case, there were opportunities for respondents to give details or otherwise explain their answers. Questions were thus accompanied by a series of optional probes to stimulate conversation. Probes helped remind respondents to describe details about hyenas in relation to their life experiences, including livelihoods, physical safety, mental health, spirituality, public health, and more. Examples of LOIs and field forms, including probes, for focus group discussions and interviews are available as Supplementary Materials.

The five questions above were generated based on the research objectives. The questions were intended to be general in scope and vetted by the research team prior to field work, with prominent input from three team members who had considerable experience conducting social surveys in Ethiopia. We were unable to pilot test the questions pre-travel given uncertainties in the precise identification of target populations and an inability to support the associated field logistics. The overall approach for problem framing and data analysis for the social science work was empirically based and, hence, non-theoretical. Text generated from focus groups and interviews was studied and synthesized by the lead social scientist with respect to the main points generated. Inductive reasoning thus prevailed in this exploratory case study. 

### 2.2. Hyena Data Collection

All data were collected after approvals were received by Utah State University’s IACUC (IACUC-2786) and written approvals by the Ethiopian Wildlife Conservation Authority. We first observed hyenas opportunistically and conducted playback surveys from several locations to estimate the number of clans and population sizes of hyenas in each city, although the survey for Addis Ababa was more limited in scope. In Addis Ababa, we sampled in three of four cardinal directions along the edge of the city based on information provided by residents. We only conducted playbacks at night to best match the active period for hyenas [37,44]. We recorded the date, time, location, and dominant habitat at each playback site; all raw data are available in the Supplementary Files. Habitat was standardized to three categories: Open area, forest, and human settlement. Due to the short time in which playbacks occurred, weather conditions were similar across sites (i.e., low to no wind and moderate temperature) and did not need to be considered further. We played a two-minute recording five times in a row of hyenas calling and whooping on a speaker (OontZ Angle3XL by Cambridge SoundWorks) before counting the number of hyenas. During each 10-minute playback period, we moved the speaker in a slow circle to increase the detection of hyenas from all directions. Each playback period was followed by a 10-minute break and the number of hyenas was counted at the end of the break. The process was repeated a second time before we moved to a new location or ended playback surveys for the night. We used night-vision binoculars (Night Owl Optics Explorer Pro 5x Night Vision Binocular) to count hyenas, and recorded all species approaching during playbacks. We used a bright flashlight to rapidly scan for eye-shine at least once at each playback survey to double-check our counts. By minimizing the use of lights, so that more skittish animals would still be detected, we were unable to appropriately determine individuals, age, or sex of all hyenas. We typically surveyed several sites on any given night in each city but separated sites to avoid double counting the same animals (e.g., [45]). 

We compared latency to approach a novel object and problem-solving skills of spotted hyenas across study sites using traditional puzzle boxes [46,47,48]. We placed two identical puzzle boxes approximately 10 m from one another in open spaces within each city. Each puzzle box was 50.8 cm^3^ in size and made of steel wire material, so that the meat reward could be seen and smelled but only obtained through the open door. The only difference between the two puzzle boxes was that the door was open to one and closed for the other, with a knotted rope (~25.4 cm long) attached to the closed door that a hyena would need to pull to open the door. The one with the open door was used to facilitate investigation of the closed puzzle box and contained less raw meat, equal to about one-third the quantity in the closed puzzle box, to assist with motivating hyenas to interact with the closed box before they were satiated. All raw meat was obtained fresh from a city butcher and consisted of remaining meat scraps and bones from meat processed that day. Latency to approach was measured for the first hyena, alone or with others, to approach either puzzle box. We recorded the time to approach within 1 m and to consume meat from the open puzzle box. We then measured problem-solving abilities by determining whether a hyena was able to successfully solve the puzzle box. This was defined as opening and consuming food from the closed puzzle box. We analyzed the latency to approach and touch the puzzle boxes using Cox proportional hazard models in Program R with the “survival” package [49]. The location and the number of hyenas were predictor variables in our models. We specified right-censored latencies for any trials where a hyena never touched the puzzle box.

We measured boldness towards humans via flight initiation tests. One researcher (JY) walked in silence along a straight line towards a hyena or group of hyenas at a normal walking speed (~3–4 km/h) during nighttime and crepuscular hours. The distance at which the hyena or group of hyenas responded (both for vigilance to walker and fleeing) was recorded by a second person (GY) observing the flight initiation test from a parked vehicle. Although we wanted to start all flight initiation distance test tests with hyenas in the same behavioral state (i.e., all at rest or all active), this presented a challenge due to high levels of other human-related activity, such as vehicles and roaming dogs. Instead, we recorded whether the hyena(s) were at rest or not before the trial began and used this factor in our analysis. We analyzed the flight initiation distance test using an ANCOVA test. 

## 3. Results

### 3.1. Human Surveys

Across the three study areas of Arba Minch, Harar, and Mekelle, 163 people contributed information. This included 15 focus group discussions, with an average of ten people each, and eight interviews (Table S1). Both focus group discussions and interviews took about an hour to complete. Participants in focus group discussions ranged in age from 18 to 72. Participants were diverse in socioeconomic aspects. As intended, the focus group discussions variously represented men, women, Muslims, Christians, young, old, poor, and the middle class. People resided in urban, peri-urban, and rural areas. Participants from peri-urban or rural locales were typically small-scale farmers. Urbanites were often semi-skilled and employed in a wide variety of occupations. Key informant interviews occurred with city officials, public health specialists, police officers, and veterinarians. All were men. An elderly man, who had hand-fed hyenas for decades in Harar as part of tourist exhibitions, was also interviewed. In retrospect, there was no apparent problems in the implementation of the focus groups or interviews caused by our lack of pilot testing questions. 

Hyenas were acknowledged by most respondents for their key roles as scavengers and sanitation agents in all study sites. City officials in Mekelle and Harar saw hyenas as important stakeholders in public health management. The ability of hyenas to quickly dispose of large carcasses (i.e., donkeys, horses, cattle) was noted as especially vital. Respondents mentioned that hyenas can also affect agricultural systems in positive or negative ways at Mekelle and Harar. For example, hyenas were reported to damage stands of mature cereal crops when engaged in vigorous nocturnal activities. The hyena presence, however, was also noted for providing security for high-value crops (i.e., Khat—*Catha edulis*) by keeping away thieves and foraging wild herbivores. 

While hyenas were said to rarely attack people overall, some respondents felt that hyenas are potentially dangerous and can maim and kill children or adults. Respondents said hyenas attack and kill livestock, although in general this was also seen as uncommon. When refuse and other scavenging resources are abundant, respondents thought that hyenas do not need to kill livestock. In general, hyenas were not viewed as having major or direct effects on income generation. One exception, however, is in Harar, as hand-feeding of hyenas in the Old City (“jugol”) has long been a revenue-generating tourist attraction for a few individuals. 

Hyenas are generally viewed as evil elements of the spiritual world in all study areas. Hyenas were regarded as the “Horse of Satan” or agents of “buda” (e.g., the “evil eye”). In terms of *buda*, certain people can be transformed into hyenas and create trouble by casting spells and doing other bad deeds. Hyenas were also viewed as clever adversaries in daily life; if you break the trust you have with hyenas by harassing them, they can take revenge on you, your family, or your livestock. Therefore, hyenas were regarded as enforcers of social norms. For example, if you do not regularly attend church in Mekelle, hyenas may visit you and create problems. Views concerning hyenas as spiritual agents of evil were commonly offered by a very wide range of people from young, educated urbanites to very old, uneducated farmers. 

Harar was the only location, however, where a very positive spiritual role is seen for hyenas as hunters of evil spirits called *jinn. Jinn* are viewed as an ever-present threat to all in terms of their ability to possess people’s souls and do bad deeds. Some respondents in Harar noted that if hyenas were to disappear, *jinn* would take over the city. Hyenas therefore must be promoted as spiritual guardians in Harar according to respondents. Harar respondents also differentiated local from “foreign” hyenas in their midst. For example, a child was killed by a hyena a few years ago in Harar, but it was rationalized that this particular hyena was not from Harar, but from somewhere else. It was not clear how residents differentiate local from “foreign” hyenas.

For Arba Minch, the general profile of hyenas in the lives of respondents was different from responses in Mekelle or Harar. City officials and professionals in Arba Minch recognized few, if any, important roles of hyenas in urban or peri-urban life. In general, the regular citizens espoused more negative or neutral views about hyenas; they have rarely directly engaged with them and typically see hyenas more as a nuisance animal. Deeper cultural connections to hyenas (i.e., *buda*) were only occasionally noted by respondents in Arba Minch. 

In each study area, people said they learned how to behave around hyenas based on life experiences. They learn locally relevant hyena etiquette from elders, family members, and friends. An example is harassing hyenas; while throwing rocks at hyenas is accepted practice in Arba Minch, it is less acceptable in Mekelle or Harar due to the pervasive views concerning *buda* or *jinn* as noted. While safely passing hyenas on a narrow urban street at night is a common skill needed for Harar, it was not acquired by respondents in Arba Minch.

Respondents in Mekelle and Arba Minch felt that their hyena populations have declined, mostly due to habitat loss. Habitat loss is primarily due to the expansion of farming that eliminates hyena denning sites in proximity to urban areas. While hyenas living in Nech Sar National Park—adjacent to Arba Minch—appear to have a haven, it was noted that hyenas in the park can be killed by pastoralists also residing in the park if hyena prey on their livestock. Safe havens for hyenas in Harar and Mekelle included land holdings of the Orthodox Christian Church; these large, forested parcels serve as cemeteries and places of meditation by priests and are protected from development pressures.

Finally, hyenas were not implicated as important agents of disease transmission at any study area, despite evidence from elsewhere that hyenas can be hosts for several virulent zoonotic afflictions [50]. City officials and health professionals generally noted instead that the management of free-roaming dogs was the main concern for public health. A similar concern was noted regarding urban leopards in India [19].

According to city officials, none of the three study areas has a formal hyena management plan. Harar has an implicit plan of sorts as the city administration wants to promote hyenas for cultural and tourism reasons. Officials noted that the lack of coordination among local agencies, however, makes hyena management a challenge in Harar. Mekelle officials acknowledged the key role of hyenas in urban sanitation, but this has not been incorporated into a plan. City officials at Arba Minch, in contrast, do not see any need for hyena-related matters in their planning agendas. Officials in each city acknowledged that plans are underway to modernize solid waste management; this includes improving the efficiency of residential and business garbage collection as well as promoting sanitary landfill development.

### 3.2. Hyena Survey, Puzzle Box, and Flight Initiation Distance Tests

We conducted playbacks between 22h00 and 02h00. A total of 215 spotted hyenas responded to 14 playback stations (Table S2). Because we attempted to distribute playback stations evenly across each city, there was a different total number of playback locations within each city (Harar n = 4; Mekelle n = 5; Addis Ababa n = 10; Arba Minch = 3). Playbacks were primarily used to determine if hyenas occupied the cities in which we had selected for the study. Hyenas responded to playbacks in all cities except Arba Minch. The hyenas were found more frequently in open area habitats than in the forest and human settlement habitats, but we did not design the survey locations to statistically evaluate habitat differences [41].

There were two hyena feeding sites related to tourism in Harar, each visited by a discrete clan, and at least three garbage collection areas where hyenas forage. We observed hyenas most commonly interacting with people (with and without livestock), free-roaming cats and dogs, and less often with wildlife, in and around Harar. Community members also showed us the gradual disappearance of hyena den sites in the surrounding valley due to the unplanned expansion of housing and agriculture. We conducted playbacks on five nights in Harar, repeating the survey at each site on two different nights. We detected hyenas at 75% of playback events and found that at least four clans occupy Harar. We estimated at least 30 hyenas occupied the Harar study site; the maximum number of hyenas counted during any one count was 21. 

In Mekelle, we observed hyenas resting during daylight hours in church forests and other vegetation around the city’s edges. We observed hyenas walking near people who were with and without livestock, golden jackals, and domestic dogs. We also observed at least one hyena clan foraging at the city landfill and near den sites in areas where human foot traffic with and without livestock is high. We only observed one historical denning area that is likely still in use. We conducted playback on five nights in Mekelle, repeating the survey at each site on two different nights. We detected hyenas at 80% of playback events and found at least five clans access parts of Mekelle, for a total of at least 82 hyenas. The maximum number of hyenas counted at one playback count was 32. At one site, a golden jackal responded to the survey. 

In Addis Ababa, we also observed hyenas interacting with people and their dogs. We observed interactions between hyenas and free-roaming dogs but did not observe any interactions involving people with livestock and hyenas. We conducted playback surveys on two nights in Addis Ababa but did not repeat a survey at any given site. We detected hyenas at 70% of playback events and found five to seven clans occurring in or around the city; in some cases, we were unsure if hyenas were from the same or different clans and therefore estimated a range in the number of clans. We estimated a minimum of 103 hyenas. The maximum number of hyenas counted during any one playback count was 19. We were unable to conduct as many playback surveys throughout the city as desired because of logistics concerning the density of humans and time. 

Hyenas were only seen in one instance on the outskirts of Arba Minch with all other sightings occurring within Nech Sar National Park. We conducted playback surveys for three nights but did not find evidence of hyenas in the city of Arba Minch, so we excluded this site for subsequent analysis of hyena behavior. However, because hyenas historically occurred within the city, social survey work was conducted.

We conducted trials with puzzle boxes during three nights in Harar and two nights in Mekelle (Table S3). We did not conduct puzzle box trials in Addis Ababa because we could not find sites without a high number of humans and dogs. There was no significant difference in latency to approach (Wald test: z = 3.49, *p* = 0.3) or touch (Wald test: z = 1.23, *p* = 0.7) the puzzle box between hyenas in Harar and Mekelle when the number of hyenas present was included in the models. However, hyenas in Harar approached sooner when the model only considered location (Wald test: z = 3.06, *p* = 0.08). 

In Harar, the puzzle boxes were tested at three different locations to obtain data from at least three discrete clans. One site was used only on the first night, a second site was used on the first and third night, and the third site was used on the second and third nights. All of the puzzle boxes were approached by hyenas with an average latency to approach time of 338.3 ± 151.5 s (n = 6). Latency until hyenas consumed meat from the open puzzle box was 1385.2 ± 384.2 s (n = 5). Hyenas did not consume meat during one puzzle box trial. Hyenas were able to open the closed puzzle box and consume meat from it on one occasion. This was the second time the site had been used during the puzzle box trial, during the third and final night, and it is likely some of the same hyenas visited the previous night; however, we were unable to confirm the identity of individuals during the trials. During this trial, the hyena opened the puzzle box 1701 s after approaching the puzzle box. A second puzzle box was unlatched but not opened on night two at a different site; hyenas started to interact with the closed box, a group of people passed by and threw rocks at the hyenas. Thus, we were unable to obtain an accurate time on latency to solve because the hyenas abandoned the site before consuming any meat from the solved puzzle box; the door was left ajar.

**Figure 2:**
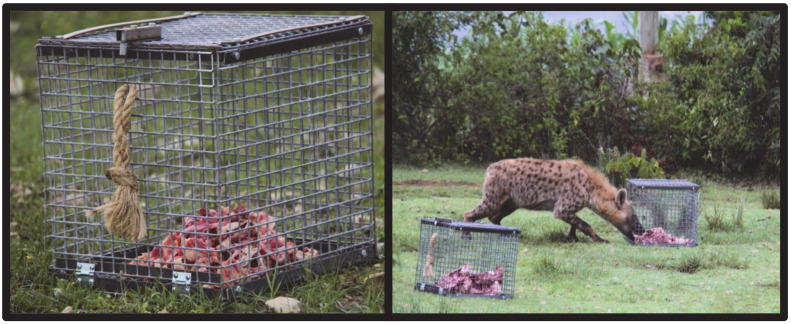
Newly baited puzzle box with door, showing the twisted knot handle that opens the door when pulled. Spotted hyena obtaining raw meat bait from puzzle box without door, with closed puzzle box in the foreground.

In Mekelle, puzzle boxes were placed at two sites, one sight per night to obtain data from at least two discrete clans (Figure 2). Three individuals or groups of hyenas approached on the first night and two on the second night. The average latency to approach was 1156.4 ± 452.8 s (n = 5). In only one instance did hyenas, in a group of three, consume food from the open puzzle box; this was 3133 s after they approached. No hyenas opened the closed puzzle box.

**Figure 3:**
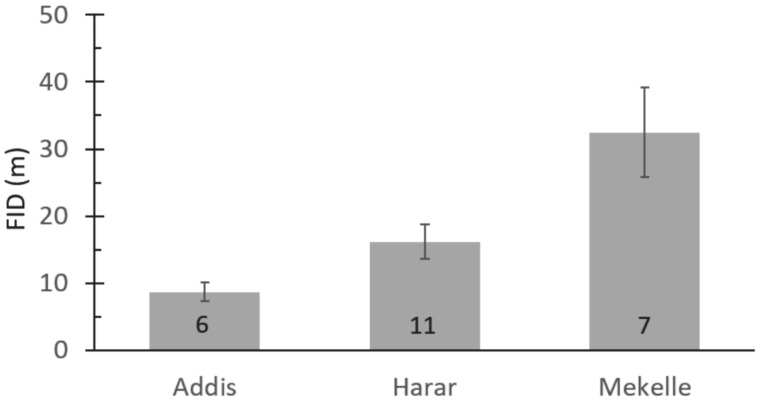
Average (± SE) flight initiation distances of spotted hyenas in three Ethiopian cities, with sample sizes shown inside the bars. FID: Flight Initiation Distance.

We completed flight initiation tests 11 times in Harar, seven times in Mekelle, and six times in Addis Ababa (Table S4). Many other attempted trials were not completed due to external disturbances (e.g., a car driving by or a dog walking up). Removing trials with external disturbances resulted in only trials conducted after dark being used in analysis. We found a significant difference in flight initiation tests among the three urban sites (Figure 3; F2,21 = 7.926, *p* = 0.00273), with hyenas initiating flight at the longest distances in Mekelle, followed by Harar. Pairwise comparisons via Tukey’s multiple comparison tests revealed significant differences between Mekelle–Addis (*p* = 0.0028) and Mekelle–Harar (*p* = 0.018), but not between Harar–Addis (*p* = 0.399). There was no effect of active versus at rest behavior before the flight initiation test (*p* = 0.66) or an interaction effect between pre-test behavior and site (*p* = 0.96).

## 4. Discussion

**Figure 4:**
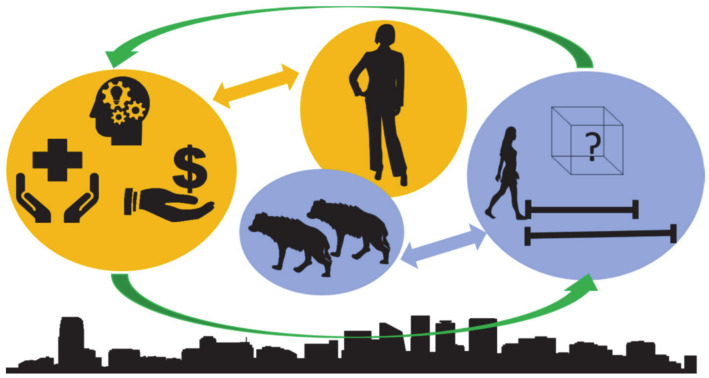
Conceptual model of hyena dynamics, human dynamics, and where they influence one another. In urban areas where humans and hyenas overlap (center), humans integrate economics, perceptions and beliefs, and public health, whereas hyenas integrate behavioral responses to humans (e.g., flight initiation distance tests) and problem-solving abilities (e.g., puzzle boxes). How both outwardly behave in response to these factors further influences behavior.

Our case study includes data on human perceptions and beliefs and hyena behavior in urban areas of Ethiopia to illustrate the types of data that could be collected to assess linkages between the two disciplines. The qualitative, exploratory approach used for collecting social science information in this study broadly confirmed other literature noting that hyenas are (1) important urban and peri-urban scavengers and sanitation agents [10,21], (2) largely insignificant predators on livestock or people in urban and peri-urban areas [21,33], and (3) often very important in the spiritual lives of citizens [21,35,51,52]. Our quantitative data on hyena presence and behavior corroborated that they (1) occupy urban areas of Ethiopia [53,54], (2) adaptively respond to human approach [55], and (3) vary in problem-solving ability [48]. The social science data have limitations, however, with respect to imbalances in the types and numbers of people engaged at each study site. Capturing specific quotations in the discourse may have better illuminated some details of hyena–human interactions. Despite such challenges, the results provide insight into the diverse human dimensions of human–hyena co-existence. Preliminary results suggest that integrating these two seemingly disparate fields of study may help explain observed patterns. However, we only collected pilot data for both disciplines and more information is still needed to understand the strength of potential feedback loops (Figure 4). A next step would be to create models that best integrate human and hyena data, but our case study lacked enough data to do so. 

We found the most uniformly positive attitudes towards hyenas among Harar residents, followed distantly by Mekelle residents (slightly positive to negative), and Arba Minch residents (neutral to negative). These rankings were merely intended to be illustrative and were achieved from an empirical synthesis of main points raised at each study site, both in terms of intensity and frequency of mention in what was admittingly a small sample. Formation of social attitudes as a function of values, beliefs, and social norms [56] may be a fruitful area for future theory formation and deductive hypothesis testing via randomly assigned social surveys (see below). 

The difference in attitudes towards hyenas also reflected in hyena behavior, whereby hyenas seemed to allow humans to approach more closely in Harar and Addis Ababa than in Mekelle. Indeed, in Mekelle, where human attitudes and perceptions were strongly linked with negative connotations, hyenas fled at the farthest distances during flight initiation tests. While hyenas largely do not occur in Arba Minch, they are observed near the edge of town and it is possible this spatial pattern is related to human views towards hyenas; they were the most negative among people we interviewed. Finally, hyenas in Harar were more apt to solve puzzle boxes, illustrating a potential difference in cognitive abilities with hyenas residing in centers with different human attitudes and beliefs. While carnivores have been shown to adapt to human behavior and therefore co-occur (e.g., [57]), we suggest including human beliefs and perceptions into considerations regarding the interactions between humans and hyenas to best explain the different responses of hyenas to our flight initiation tests and puzzle box trials and outcomes of coexistence. However, our case study was correlative and future studies should explore causative mechanisms for how humans shape urban carnivore behavior.

If the associations between hyena behavior and human behaviors and perceptions are real, it is interesting that there was no significant difference in the flight initiation test between Harar and Addis Ababa. Although we were unable to collect survey data from community members in Addis Ababa, our observations indicated the residents are likely the most disconnected to carnivores among the residents in the four cities. It could also be that hyenas in Addis Ababa encounter humans at such a high rate that they have become habituated to their presence and the majority of humans likely pose no threat because government policy generally prohibits the killing of hyenas and other wildlife [58]. In fact, we were unable to set up puzzle boxes in Addis Ababa because we could not find areas known to be used by hyenas based on our playback surveys that would not attract attention of humans or free-roaming dogs. This is in contrast to Harar, where hyenas may have realized that humans pose no threat across less frequent encounter rates. This suggests learning that may have been facilitated with many hyenas also being fed at one of the two tourist feeding stations in Harar. At the same time, hyenas in Harar but not Mekelle solved the puzzle box in the short amount of time trials were conducted. Hyenas may be more willing to approach a novel object in Harar and have greater cognitive capacity—a trait that may help them survive in a highly complex human environment. This could be explained by the long history of interactions with humans in Harar relative to that for Mekelle.

We suspect the drivers of the relationship revolve around local food supplies for hyenas and ecosystem services needed in communities [59]. It can be hypothesized that when the net benefits of hyenas to human society are high, hyenas will be tolerated but when net benefits are low, hyenas will not be tolerated. Such perspectives could be incorporated in the analysis of variation in social attitudes towards hyenas [56]. We found that hyenas are perceived to provide a regulating service to people in the form of promoting urban sanitation in Harar and Mekelle, a cultural service in the form of spiritual support as consumers of *jinn* in Harar, provisioning services in terms of crop protection in Mekelle and Harar, and of income generation from hyena feeding exhibitions for tourists in Harar. These findings are similar to services provided by urban leopards [19,29]. The abundance of anthropogenic food sources in urban and peri-urban areas—along with human behaviors and policies that accommodate or tolerate hyena presence—have facilitated human tolerance that allows the coexistence of hyenas and humans in some of these cities. Human tolerance of hyenas has previously been attributed to beliefs that hyenas consume *jinn* in the Harari Region [51] as well as to the abundance of anthropogenic foods that discourage hyenas from preying on livestock [21,34].

While tolerance may be observed in most cities, the negative effects of hyenas on humans still occur and likely also create a feedback loop that reinforces how hyena behavioral traits emerge. We found negative effects to include occasional livestock depredation and attacks on people, and the specter of crop damage in Mekelle or Harar. The role of *buda* can also be viewed as largely negative on people’s mental well-being at Mekelle and Harar, with a trace evident at Arba Minch. 

Spiritual beliefs merited the most attention overall in the formation of behaviors and attitudes about hyenas. The cultural importance of *jinn* was central to the prevailing positive attitudes about hyenas in Harar; one core belief is that when hyenas whoop at night, it indicates that *jinn* are being eaten by hyenas and thus people’s souls are protected from possession. Although the *jinn* concept is often associated with Muslim culture, it may pre-date Islam [21,35]. In contrast to *jinn*, the *buda* concept involving hyenas is found in the Amhara Orthodox Christian culture of the highlands, as well as in the Oromo culture of the southern region [21,35]. The distinctions between *jinn* and *buda* may translate into the different cultural valuations noted by respondents in different cities, with hyenas seen as spiritual saviors in Harar versus an evil being in Mekelle. Arba Minch respondents, in contrast, revealed more moderate views concerning beliefs in *buda* or *jinn.* However, larger samples of respondents using qualitative and quantitative research are needed to confirm these patterns. 

Environmental history and land use play large roles in shaping contemporary hyena–human interactions in Ethiopia [21] and likely shaped responses from respondents among the cities. The regional expansion of the human population over centuries has eliminated natural habitats and prey that traditionally supported large carnivores such as hyenas. The need for hyenas to then scavenge urban refuse and kill livestock to replace native food sources has nurtured both positive and negative interactions with people [21]. People hand-feeding hyenas has long been an aspect of hyena–human relations in Harar, for example, and has contributed to coexistence there [35,52]. Information we gathered from respondents in Harar and Mekelle about their local landscapes and future development plans revealed a tenuous situation for maintaining hyena populations; local human population growth and expansion of suburbs and agriculture threaten hyena denning sites in both locales. 

We speculated that the Arba Minch situation differs from that of Mekelle or Harar because of one key factor, namely hyenas in the Arba Minch area never fully lost access to natural prey resources because of the creation of Nech Sar National Park adjacent to the city. Hyenas have thus had less need to forage in Arba Minch town as it has grown, and hence hyena–human relationships have largely remained random or undeveloped. A relative lack of direct hyena–human interactions over time has not fostered the intense cultural and other systemic attributes that characterize the Harar situation, for example. 

After discussions with community members in Arba Minch, we determined that our lack of detection was likely a valid account of hyena occupancy. We found hyenas near the edge of town and in the neighboring Nech Sar National Park, suggesting hyenas do not often enter the city center. We found enough evidence of hyenas in the other three cities to support the idea that populations of hyenas exist in each. These findings support previous research showing hyenas living in Ethiopian cities [34]. 

Preliminary data suggest hyenas fled at greater distances in Mekelle than in Harar or Addis Ababa during flight initiation tests. Although the trend was for hyenas to remain in place at the closest distance in Addis Ababa, this was not significantly different from the Harar flight initiation test. It is unclear if hyenas are responding similarly or if this is an artifact of small sample sizes. Small sample sizes prevented us from including possible confounding variables in analysis or move beyond a preliminary analysis. We attempted to obtain larger sample sizes in each city, but tests were often disrupted by the appearance of vehicles, especially in Addis Ababa, or free-roaming dogs. This is a challenge with research in urban areas but also may explain why the hyenas allowed a human to approach at such a close distance before fleeing. Hyenas likely encounter people each day and, in most cases, are unlikely to see humans as threatening [60]. Overall, the distances were all extremely close and a bit uncomfortable for the human walking towards the hyenas in the dark. In all cases, the hyenas eventually fled and did not respond with any aggression. While flight initiation tests have not previously been conducted on hyenas for comparison to our results, they have been conducted on at least one other urban carnivore, coyotes. In that study, rural and urban coyotes were compared, and we note here that both fled at much farther distances than the hyenas [16]. Coyotes are hunted in western rural areas of the USA where the study occurred and face lethal removal in response to urban conflicts. Further research should obtain more data on hyenas in urban areas, compare urban hyenas to those in more remote landscapes, and better control for additional confounding factors.

The puzzle box tests were conducted in a short amount of trials relative to another puzzle box tests with hyenas [48], so, unsurprisingly, we found less success at solving the puzzle box than this previous study. However, we were able to obtain important information from these trials, most notably the time to approach a novel object and that only hyenas in Harar could successfully solve the puzzle box under these conditions. Interestingly, there are many free-roaming cats (*Felis catus*) in Harar, and some were attracted to our puzzle box. They ate from the open box and kept the hyenas from accessing the puzzle boxes during one trial. Those data were removed from the analyses, but intimate that hyenas are also navigating co-occurring with domestic pets as well as human owners. Domestic cats can be killed by carnivores, often noted as a source of conflict in urban areas [61]. Notably in our observation, three cats dominated the food supply and the seven hyenas. More data on puzzle box tests of urban hyenas, comparing them to hyenas in more remote landscapes, and controlling for potentially confounding factors (i.e., age, sex, prior experience), are needed.

## 5. Conclusions

Our case study exemplified the methodology for understanding these potential feedbacks between human and carnivore systems (Figure 4). More research on how positive and negative effects of hyenas on humans and humans on hyenas is needed to assess the possible existence and to measure the strength of the potential feedback loops. While we lacked enough data to take this final step, our case study highlighted how integrated relationships can be studied. Understanding systems where humans and large carnivores coexist is important, especially because these shared landscapes are rapidly changing. For example, our findings that peri-urban hyena denning sites are often at risk, and that at least three of our study areas plan to modernize refuse management systems, have important and negative implications for urban hyenas which in turn could impact human interactions with hyenas. As many parts of the globe develop and modernize, carnivore–human interactions will likely be altered unless accounted for in development planning.

## Supplementary Materials

The following are available online at https://www.mdpi.com/2076-2615/10/12/2400/s1, Tables S1–S4: Raw data files.
